# Body Mass Index is Associated with blood pressure and vital capacity in medical students

**DOI:** 10.1186/s12944-023-01920-1

**Published:** 2023-10-18

**Authors:** Lingxia Song, Jiajin Li, Sen Yu, Yunjia Cai, Huan He, Jiayi Lun, Li Zheng, Jufeng Ye

**Affiliations:** 1 School of Public Health, Southern Medical University, Guangzhou, 510515 Guangdong China; 2 Department of Epidemiology, School of Public Health, Southern Medical University, Guangzhou, Guangdong China; 3 Experimental Teaching Center of Preventive Medicine, School of Public Health, Southern Medical University, Guangzhou, Guangdong China

**Keywords:** Body mass index, Blood pressure, Vital capacity, Medical students

## Abstract

**Background:**

The widely reported associations between body mass index (BMI) and various chronic diseases, such as hypertension and asthma, have garnered significant attention. Nonetheless, there remains a dearth of research dedicated to understanding the health impacts of medical school on the students, who experience considerable academic pressure. In that context, this study was driven by the goal of investigating the intricate interplay between BMI, blood pressure (BP), and vital capacity among medical students.

**Methods:**

This study included a cohort of 843 medical students enrolled at Southern Medical University who were selected through random cluster sampling. Within this cohort, measurements of height, weight, BP, and vital capacity were taken. Subsequently, both BMI and vital capacity index (VCI) were calculated for each participant. By categorizing the subjects into four groups according to BMI classifications, a comprehensive analysis that included correlation assessments and binomial logistic regression was conducted.

**Results:**

Within the participant pool, 9.4% and 3.8% of participants were classified as overweight and obese, respectively. Additionally, the prevalence of prehypertension, hypertension, and poor VCI was 18.1%, 2.7%, and 13.5%, respectively. Notably, male students exhibited a higher prevalence of the aforementioned health issues than their female counterparts. Correlation analysis revealed that BMI displayed positive associations with systolic blood pressure (SBP), diastolic blood pressure (DBP), and vital capacity (r = 0.372, 0.257, 0.428; *P* < 0.001). However, an inverse correlation emerged between BMI and VCI (r = -0.284, *P* < 0.001). Further analysis revealed that overweight and obese individuals faced an elevated risk of high blood pressure ([OR 2.05, 95% CI 1.15–3.67] and [OR 5.44, 95% CI 2.28–13.02], respectively) compared to their normal-weight counterparts. Moreover, these groups also exhibited a higher risk of poor VCI ([OR 5.25, 95% CI 3.04–9.06] and [OR 15.61, 95% CI 6.81–35.81], respectively), while underweight subjects experienced a reduced risk ([OR 0.19, 95% CI 0.07–0.52]).

**Conclusions:**

BMI demonstrated a notably strong positive correlation with both BP and vital capacity and a negative correlation with VCI. Therefore, for medical students as well as the daily health care of patients, weight control is recommended to better combat obesity-related diseases, for example, cardiopulmonary diseases, gout and diabetes.

## Introduction

Obesity has reached pandemic proportions worldwide over the past five decades [1]. According to the World Health Organization, the global prevalence of adults with a body mass index (BMI) exceeding 23.9 kg/m^2^ increased by 27.5% from 1980 to 2013, while children experienced a staggering 47.1% increase [2, 3]. Extensive reports have linked high BMI to an elevated risk of various diseases, such as diabetes [4], cardiovascular disease [5], cancer [6], and musculoskeletal disorders [7], all of which adversely affect the overall quality of life. Notably, the incidence of hypertension all around the world was calculated as 25% within adult people in 2020, with projections indicating a rise to 29% by 2025 [8]. Moreover, it has been observed that obesity contributes to 60–70% of hypertension cases, with the obese population facing a 3–4 times higher risk than individuals with a normal weight [9]. Additionally, obesity-associated fat accumulation can lead to an increase in chest wall thickness, resulting in heightened respiratory resistance and compromised lung function, as indicated by reduced vital capacity [10]. As such, it is imperative to conduct an in-depth investigation into the complex and intricate correlation between BMI, blood pressure (BP), and vital capacity.

Numerous studies have expressed a shared concern that the issue of hypertension is expected to be more serious among children and adolescents, largely attributed to the increasing trend of obesity. Notably, a cohort study involving medical students revealed a notable association between BMI and the risk of hypertension, emphasizing that even modest weight gain during youth could significantly elevate the likelihood of developing hypertension later in life [11]. Moreover, findings from another cohort study conducted in Israel underscored the incremental nature of this relationship, with a unit increase in BMI correlating with a heightened risk of hypertension [12]. These collective findings establish a clear and substantial connection between BMI and hypertension to a considerable extent. However, the applicability of these observations to Chinese medical students remains to be ascertained.

Furthermore, the recent literature regarding the correlation between BMI and vital capacity exhibit inconsistency. While some studies, such as the work by Littleton SW, have reported reduced lung capacity among the obese population in comparison to individuals with normal weight [13], contrasting results were observed in a survey of Tibetan students at Guangxi Medical University, which found no discernible correlation between BMI and lung capacity [14].

Therefore, recognizing the inconsistencies mentioned above, this study seeks to confirm these associations using a sample of students majoring in medicine in China. Additionally, considering the scarcity of research on the prevalence of different weight statuses within this specific demographic, this study also endeavors to examine this aspect.

## Materials and methods

### Study design and participants

**Fig. 1:**
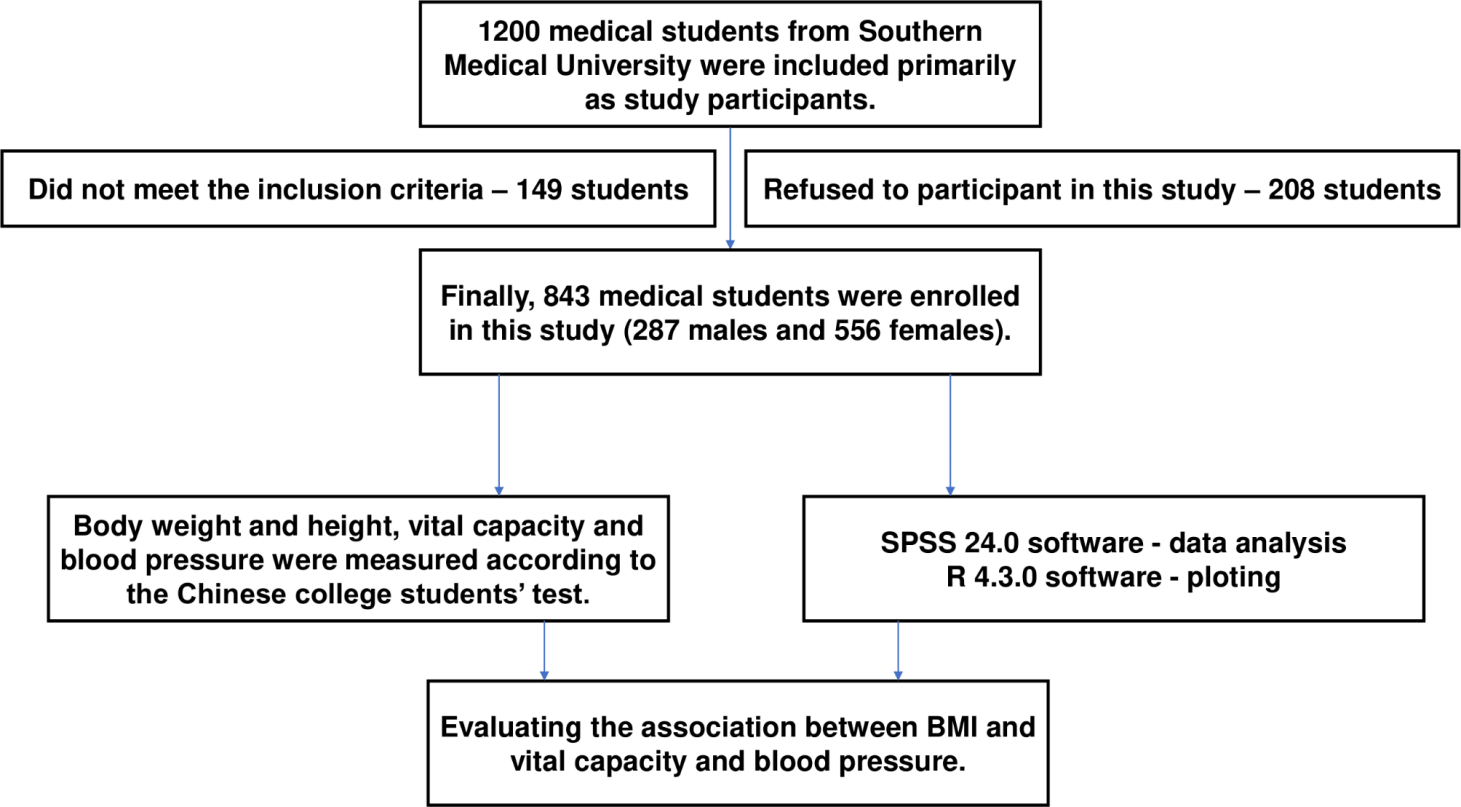
Flow chart of the study enrollment

The structure of the whole research was designed as a cross-sectional study. This study was conducted in Guangzhou, China, from November to December 2022 utilizing a cluster sampling method. Initially, a total of 1200 medical students from Southern Medical University were recruited as potential participants. Ethical approval for all data sources used throughout the entire research was obtained from The Southern Medical University Ethics Committee. Subsequently, 149 participants were not suitable to the selected criteria and 208 participants refused to join the research. Hence, all 357 individuals were excluded from the study, leaving a final cohort of 843 eligible participants (including 287 males and 556 females). The inclusion criteria stipulated that participants should be Chinese medical students in apparent good health. Participants who exhibited the following characteristics were excluded from the study: (a) students diagnosed with metabolic, cardiovascular, or respiratory diseases and (b) students who did not provide informed consent. The study enrollment process is illustrated in Fig. 1.

### General characteristics

During the cross-sectional study, a standardized face-to-face questionnaire was distributed to the enrolled medical students. This questionnaire encompassed various sections, including informed consent, sociodemographic inquiries, and closed-ended questions (yes or no) regarding medical history and results of measurement. The sociodemographic segment explored factors such as age and sex. The medical history component focused on conditions such as diabetes, chronic cardiovascular and pulmonary ailments, cancer, and gout. Participants were required to recall whether they had been diagnosed with any of the mentioned conditions since birth. The final section of the questionnaire documented the measurement outcomes for body weight, height, BP, and vital capacity. The methods employed to obtain each of these measurements are elaborated upon below.

### Anthropometric measurements

Participants’ body weight and height were measured while wearing lightweight clothing and not wearing shoes using the height and weight measurement instrument ST-2000 (5000) (ZhongTiTongFang Corporation, Jiangsu, Nanjing, China). Height measurements were recorded with an accuracy of 0.1 cm, while weight measurements also the same but the unit is kilogram. Subsequently, using the equation kg / m^2^ can obtain BMI. Following the national BMI stratification guidelines for China [3], less than 18.5 kg / m^2^ is classified as underweight; between 18.5 and 23.9 kg / m^2^ is regard as normal weight; between 23.9 and 27.9 kg / m^2^ is regard as overweight; and over 27.9 kg / m^2^ is classified as obesity. These categories encompassed the four aforementioned degrees of weight status.

### Vital capacity test

Vital capacity measurements were obtained using a spirometry meter FH-2000 (5000) (ZhongTiTongFang Corporation, Jiangsu, Nanjing, China). The procedure involved participants assuming a standing position and holding a disposable mouthpiece. Subsequently, they exhaled as forcefully as possible after taking a deep breath. Subjects should finish the protocol thirdly with a 15-second break, and the highest recorded value was considered the final result. For the definition of poor vital capacity, thresholds were set at lower than 3100 mL for male students and 2000 mL for female students. To account for the potential influence of certain anthropometric indices on vital capacity, the vital capacity index (VCI) was calculated. This index incorporates weight to provide a more reliable measure of lung ventilation function using the formula VCI = vital capacity (mL)/weight (kg). For the purpose of health risk analysis, male students with VCI < 55 mL/kg and female students with VCI < 43 mL/kg were categorized into the “poor VCI” group [15].

### Blood pressure measurement

A HEM-7136 mercury sphygmomanometer (Omron, Shanghai, China) was utilized to measure BP. Subjects were told to calm down and sit peacefully, placing their right arms comfortably at a vertical level. BP readings were taken a minimum of two times with a 5-minute interval. Two consecutive measurements were taken, and their average values were computed and recorded unless the interval between them exceeded 10 mmHg. Participants were categorized as having normal BP (SBP < 120 mmHg and DBP < 90 mmHg), prehypertension (SBP 130–139 mmHg and/or DBP 80–89 mmHg) and hypertension (SBP ≥ 140 mmHg and/or DBP ≥ 90 mmHg) [16]. To assess the risk of health problems, two groups were taken into consideration within the analysis: one included those with normal BP, while the other one included those with prehypertension or hypertension.

### Statistical analysis

Post hoc analysis was carried out to compare SBP, DBP, vital capacity, and VCI based on weight status. Boxplots told the correlation between BMI and SBP, DBP, vital capacity, and VCI. A student’s t-test was conducted to probe the heterogeneity of BMI among different health problem groups. Two multiple logistic regression models were employed in this study. Both models included adjustments for sex and age. However, Model 1 incorporated an additional adjustment for vital capacity, while Model 2 included an adjustment for blood pressure. These models were utilized to calculate the value of OR and their corresponding 95% CI for high BP and poor VCI according to weight status. Data are presented in a standard format. Substantial heterogeneity was observed as the p-value < 0.05 []. Boxplots and scatter plots were generated using R software (R 4.3.0). All the statistical procedures described were performed utilizing SPSS software (v. 24).

## Results

### General characteristics

A total of 843 individuals aged 21–24 years, comprising 287 male students and 556 female students, were included in the analysis. Table 1 presents a comprehensive comparison of various parameters between male and female students. Notably, male students exhibited higher values in multiple aspects, including height, weight, BMI, SBP, DBP, vital capacity, and VCI. Regarding the prevalence of health-related issues, the distribution was as follows: 23.5% underweight, 9.4% overweight, and 3.8% obese. Prehypertension was observed in 18.1% of participants, while 2.7% had hypertension. Additionally, 5.2% exhibited a poor vital capacity, and 13.5% had a poor VCI. A sex-based analysis indicated differences in the prevalence of weight-related problems. Specifically, underweight was more prevalent among female students (28.6% vs. 13.6%), while overweight (2.9% vs. 22.0%) and obesity (1.6% vs. 8.0%) were less common in comparison to male students. Moreover, male students showed a higher prevalence of poor vital capacity (4.1% vs. 7.3%) and poor VCI (9.5% vs. 21.3%) (p < 0.05). Table 1 provides the summarized findings for clarity.

**Table 1:** General characteristics of the participants

Characteristics	Male (n = 287)	Female (n = 556)	P value	Total (n = 843)
**Anthropometric measures**				
Age (years)	21.39 ± 0.06	21.26 ± 0.04	> 0.05	21.30 ± 0.03
Height (cm)	172.74 ± 0.38	161.49 ± 0.23	< 0.05	165.32 ± 0.27
Weight (Kg)	65.57 ± 0.72	52.34 ± 0.34	< 0.05	56.84 ± 0.40
BMI (Kg/m^2^)	21.94 ± 0.21	20.09 ± 0.12	< 0.05	20.72 ± 0.11
**Blood pressure**				
SBP (mmHg)	114.78 ± 0.64	103.19 ± 0.38	< 0.05	107.14 ± 0.38
DBP (mmHg)	72.60 ± 0.58	67.34 ± 0.36	< 0.05	69.47 ± 0.33
**Breathing capacity**				
Vital capacity (mL)	4052 ± 43.11	2783.61 ± 21.14	< 0.05	3215.43 ± 28.95
VCI (mL/Kg)	62.61 ± 0.60	53.65 ± 0.38	< 0.05	56.70 ± 0.35
**Health problems (%)**				
Underweight	13.6	28.6	< 0.05	23.5
Overweight	22.0	2.9	< 0.05	9.4
Obesity	8.0	1.6	< 0.05	3.8
Prehypertension	35.9	9.2	> 0.05	18.1
Hypertension	5.9	0.7	> 0.05	2.7
Poor vital capacity	7.3	4.1	< 0.05	5.2
Poor VCI	21.3	9.5	< 0.05	13.5

SBP: systolic blood pressure; DBP: diastolic blood pressure; VCI: vital capacity index

### Comparison of SBP, DBP, vital capacity and VCI according to weight status

**Fig. 2:**
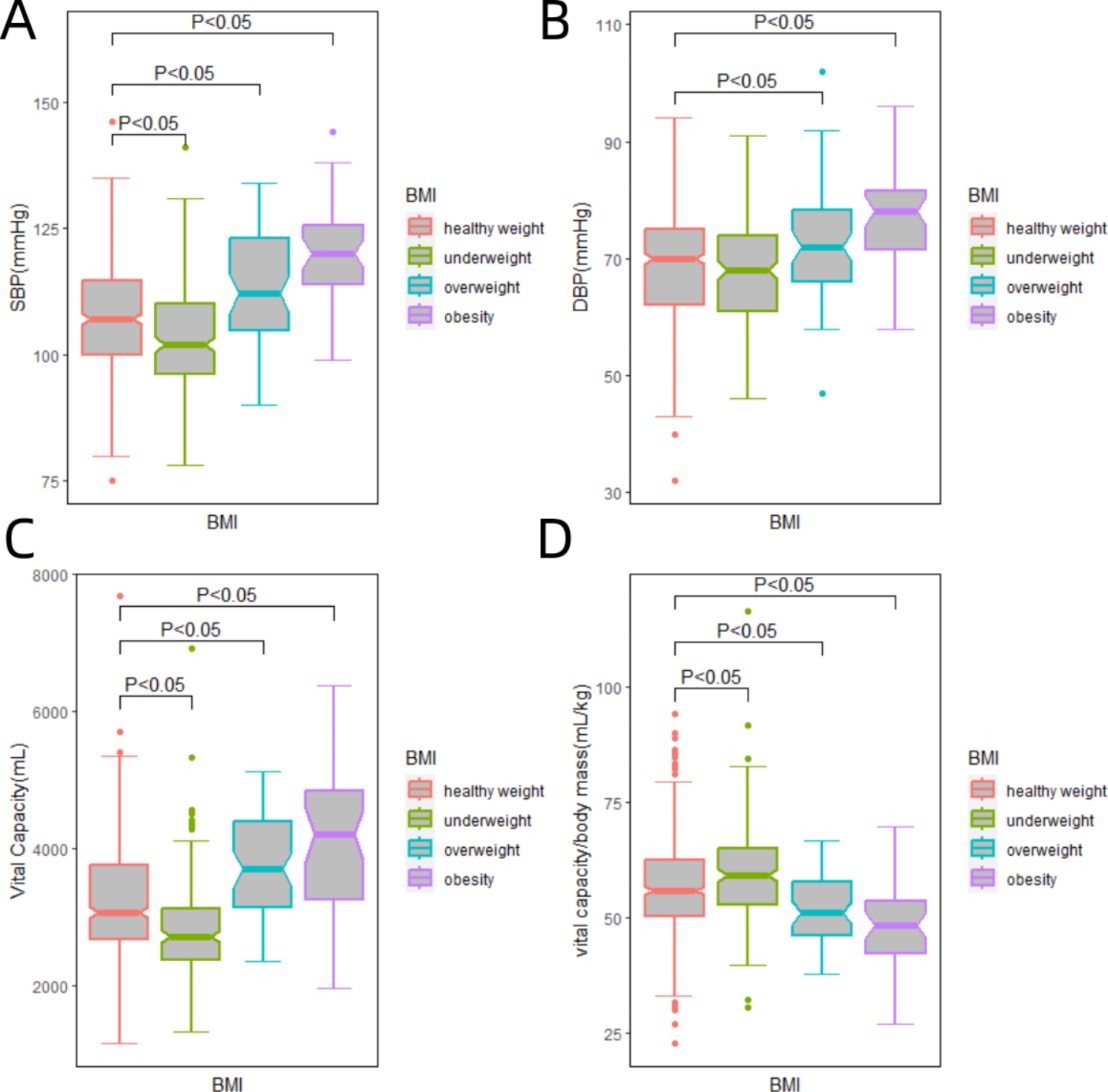
Comparison of SBP (**A**), DBP (**B**), vital capacity (**C**), and VCI (**D**) by weight status

Figure 2 illustrates notable variations in vital capacity, SBP, DBP, and VCI values among participants with diverse weight statuses. Table 2 reveals that individuals in the obesity group exhibited higher SBP, DBP, and vital capacity while manifesting lower VCI in contrast to the other groups (underweight, normal weight, and overweight). Conversely, participants in the underweight group demonstrated a contrasting pattern when compared to the other three groups.

**Table 2:** Details of Comparison on SBP, DBP, Vital Capacity, and VCI by weight status

Characteristic	Underweight (n = 198)	Normal weight (n = 534)	Overweight (n = 79)	Obesity (n = 32)	P Value
**SBP(mmHg)**	103.20 ± 0.76	106.85 ± 0.45	113.44 ± 1.31	120.69 ± 1.53	< 0.001
**DBP(mmHg)**	67.84 ± 0.63	68.98 ± 0.40	73.43 ± 1.17	77.91 ± 1.37	< 0.001
**Vital Capacity** **(mL)**	2815.56 ± 48.83	3232.94 ± 34.39	3692.76 ± 92.74	4219.03 ± 174.49	< 0.001
**VCI (mL/Kg)**	59.95 ± 0.75	56.79 ± 0.43	51.43 ± 0.90	48.06 ± 1.62	< 0.001

SBP: systolic blood pressure; DBP: diastolic blood pressure; VCI: vital capacity index

### Correlation of BMI and SBP, DBP, vital capacity and VCI

**Fig. 3:**
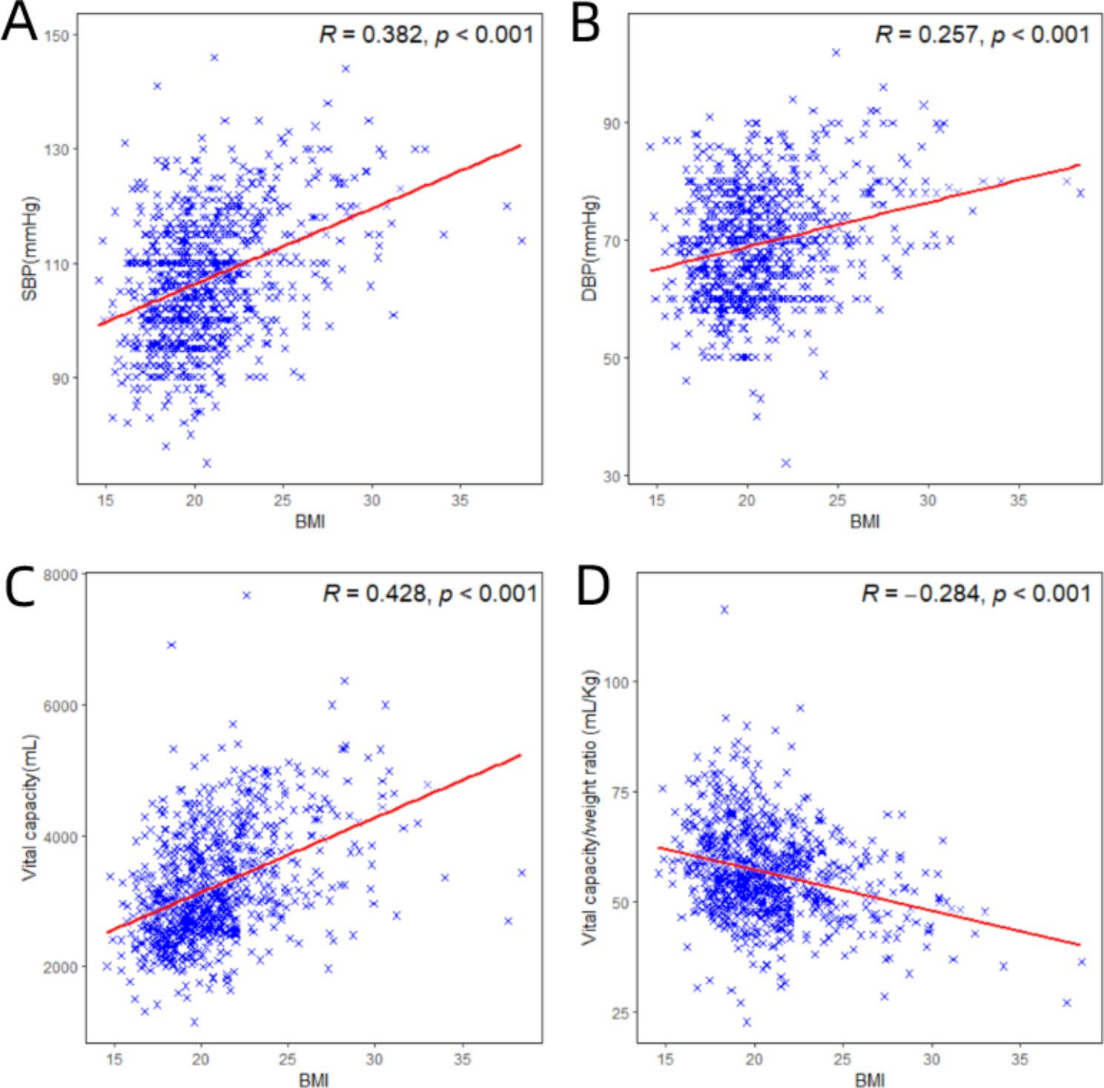
Scatter plot of the correlation between SBP (**A**), DBP (**B**), vital capacity (**C**), VCI (**D**) and BMI (**A-D**)

As illustrated in Fig. 3, Pearson’s correlation analysis was conducted, which revealed significant positive correlations between BMI and SBP, DBP, and vital capacity (r > 0, *P* < 0.001). However, VCI exhibited a negative correlation with BMI (r < 0, *P* < 0.001). In simpler terms, as BMI decreased, there was a worsening trend in vital capacity, whereas VCI demonstrated an opposite pattern.

### Comparison of weight status on the basis of health problems of prehypertension, hypertension, poor vital capacity and poor VCI

In comparison to their counterparts (as shown in Table 3), students with prehypertension, hypertension and poor VCI exhibited higher BMI values (*P* < 0.001 for all). In contrast, students with poor vital capacity demonstrated a lower BMI (*P* = 0.005).

**Table 3:** Comparison of BMI on the basis of health problems

Health problems	BMI	*P* Value
**Prehypertension** **(SBP 130–139mmHg and(or) DBP 80–89mmHg)**		
Yes	22.05 ± 0.32	<0.001
No	20.48 ± 0.11	
**Hypertension** **(SBP ≥ 140mmHg and(or) DBP ≥ 90 mmHg)**		
Yes	24.58 ± 0.81	<0.001
No	20.66 ± 0.11	
**Poor vital capacity** **(< 2100/3000 ml for female and male)**		
Yes	19.42 ± 0.38	0.005
No	20.79 ± 0.11	
**Poor VCI** **(< 43/55 mL/kg for female and male)**		
Yes	24.19 ± 0.41	<0.001
No	20.17 ± 0.09	

VCI: vital capacity index

### Comparison of the risk of high BP and poor VCI according to weight status

As shown in Table 4, the incidence of high BP increased progressively as the BMI values escalated, from 13.6% in the underweight group to 62.8% in the obesity group. A similar trend was observed for poor VCI, with prevalence rates ranging from 2.0% for the population with a BMI less than 18.5 kg/m^2^ to 69.8% for the same with a BMI over 27.9 kg/m^2^. The multivariable-adjusted analyses consistently indicated that various weight statuses actually have a significance association with the incidence of high BP and poor VCI. Compared to the normal one, the ORs for high BP were 2.05 (95% CI 1.15–3.67; *P* = 0.016) for the overweight group and 5.44 (95% CI 2.28–13.02; *P* < 0.001) for the obesity group. Similarly, weight status exhibited a graded relationship with the risk of poor VCI (OR 0.19, 95% CI 0.07–0.52 for underweight; OR 5.25, 95% CI 3.04–9.06 for overweight; OR 15.61, 95% CI 6.81–35.81 for obesity).

**Table 4:** Risk of high BP and poor VCI among college students with weight status

Weight Status	High BP (Model 1)			Poor VCI (Model 2)		
	%	OR (95% CI)	*P* Value	%	OR (95% CI)	*P V*alue
**Normal weight**	18.3	1	-	10.9	1	-
**Underweight**	13.6	0.92 (0.56–1.50)	0.726	2.0	0.19 (0.07–0.52)	< 0.001
**Overweight**	36.7	2.05 (1.15–3.67)	< 0.05	35.0	5.25 (3.04–9.06)	< 0.001
**Obesity**	62.8	5.44 (2.28–13.02)	< 0.001	69.8	15.61 (6.81–35.81)	< 0.001

Model 1: OR adjusted for age, sex and vital capacity; Model 2: OR adjusted for age, sex and blood pressure; BP: Blood pressure; VCI: vital capacity index

## Discussion

Obesity is a well-known significant risk factor for noncommunicable diseases (NCDs), such as chronic obstructive pulmonary disease (COPD) and hypertension [18]. However, the correlations between BMI and vital capacity are inconsistent, and the relationship between BMI and BP is unknown among Chinese medical students. Therefore, this study is the first to examine the prevalence of each BMI level and its association with vital capacity and BP among Chinese medical students. Interestingly, positive associations with systolic and diastolic BP were identified, and a negative relationship with VCI was found.

After rigorous statistical analysis, the proportions of underweight, overweight, and obesity were determined to be 23.5%, 9.3%, and 3.7%, respectively. Concerningly, the issue of underweight among students majoring in medicine in China was relatively more significant compared with the Africans (4.9%, 21.7% and 3.0%), consistent with earlier findings [19]. This disparity can likely be attributed to variations in dietary habits across different ethnic groups. For instance, some ethnicities demonstrate a higher intake of energy-dense, nutrient-poor snack foods [20], while the typical Mediterranean diet prioritizes the consumption of fruits and vegetables [21]. In fact, research indicates that Chinese college students tend to consume fewer daily snacks than Saudi students, and they have a higher daily intake of fruits and vegetables than African students [22]. Consequently, Chinese students are more likely to have an energy intake lower than their expenditure, leading to a reduced risk of obesity and a higher prevalence of underweight conditions.

Furthermore, the study findings indicated that male students exhibited a greater prevalence of overweight and obesity than their female counterparts. Conversely, female students demonstrated notably higher rates of underweight than male students. A plausible explanation could stem from societal sex stereotypes, wherein women are often expected to place greater emphasis on their appearance compared to men. A prior study has shown that females tend to strive for a lower BMI to enhance body esteem and alleviate anxiety [23, 24].

Previous systematic reviews and retrospective studies consistently indicated that higher BMI values are associated with elevated BP in adults, a trend that has been widely confirmed [25, 26]. This is in line with our current results, which reveals a positive correlation between BMI and all BP measurements within medical students. Notably, the risk analysis highlighted that overweight individuals and obese individuals exhibited elevated risks of 2.05 and 5.44 for hypertension, respectively, compared to individuals whose weights are in the normal range. The underlying mechanism is intricate and often interconnected. A study conducted on the Japanese population revealed that a BMI ≥ 23 serves as a risk factor for insulin resistance (IR) [27]. IR, resulting from the accumulation of excessive lipids, leads to lipid deposition in various tissues, such as blood vessels, triggering inflammation in the vicinity. Simultaneously, excess fat triggers the secretion of proinflammatory cytokines, contributing to atherogenesis. Eventually, these factors disrupt blood pressure regulation, potentially leading to elevated blood pressure or hypertension [28, 29]. Hence, it is imperative for young individuals to prioritize maintaining a lower BMI to prevent and manage hypertension effectively [30].

Both vital capacity and VCI are commonly employed in assessing lung function. Notably, VCI excels at gauging the influence of body weight on pulmonary performance and indirectly reflects respiratory function and maximal oxygen consumption in humans [31]. The current study highlights a noteworthy rise in vital capacity with increasing BMI, indicating a positive correlation between BMI and vital capacity. However, the trend is reversed for VCI, which is consistent with previous findings [31, 32]. Moreover, overweight and obese individuals face elevated risks of poor VCI compared to those with normal BMI, with relative risks of 5.25 (95% CI: 3.04 to 9.06) and 15.61 (95% CI: 6.81 to 35.81), respectively.

Although the precise mechanism underpinning these associations remains unclear, one plausible interpretation involves excessive adipose tissue. A meta-analysis revealed a robust correlation between BMI and body fat percentage among college students [33]. As BMI increases, excess body fat accumulates, resulting in weight gain. Over time, the body adapts to this increased load, potentially leading to an eventual rise in vital capacity [34].

Considering the inverse correlation between BMI and VCI, which reflects cardiopulmonary function, a plausible explanation is the limitation of inspiratory movements due to excess adipose tissue [32]. With increasing BMI, excess fat accumulates mainly in the abdominal and thoracic areas, mainly hindering two basic inspiratory movements: diaphragmatic contractions that push abdominal contents downward and forward and rib movements that expand chest diameter. These combined factors ultimately lead to decreased lung capacities, reducing actual tissue oxygen supply and potentially impacting heart contraction. This entire process can manifest as a decline in VCI [35].

### Study strengths and limitations

Some of the advantages of this articles are worth mentioning. First, a large sample size was utilized in the analysis, enhancing the potential for generalizability to medical students. Second, anthropometric, blood pressure, and vital capacity parameters were measured following standardized protocols, thereby enhancing measurement accuracy. Importantly, this study stands as an inaugural investigation among Chinese medical students. Consequently, it can serve as a valuable reference for forthcoming research endeavors targeting other ethnic groups, particularly given the scarcity of studies focusing on this specific cohort.

However, this study had certain limitations that warrant acknowledgment. First, its cross-sectional design inherently restricted the capacity to establish causality in the relationships of BMI with blood pressure and vital capacity. However, valuable etiological insights were provided. To comprehensively discern causal pathways, longitudinal prospective investigations are imperative. Second, the study’s scope was confined solely to the Chinese population, precluding direct extrapolation of outcomes to diverse ethnic cohorts. Moreover, although BMI is not the definitive tool for measuring body fat, its applicability in large-scale studies is reasonable, as supported by numerous investigations. However, it is important to introduce updated normative values, as the original categorization inadequately reflects the nutritional status of adolescents in specific regions [36]. Furthermore, augmenting the validity of these findings necessitates additional research utilizing novel obesity-related indices for example, the visceral adiposity index [37].

## Conclusions

In summary, weight status is closely associated with blood pressure and lung function among medical college students. Preventing overweight and obesity in college students may be an effective method to avoid the future development of hypertension and decline in lung function, similar to the daily health care of patients.

## Data Availability

All data generated or analyzed during this study are included in this published article.

## Abbreviations

BMI: Body mass index
VCI: Vital capacity index
BP: Blood pressure
SBP: Systolic blood pressure
DBP: Diastolic blood pressure
NCDs: Noncommunicable diseases
COPD: Chronic obstructive pulmonary disease
IR: Insulin resistance
